# Cardiovascular fellow-in-training feedback on virtual and simulator-based learning experience during Covid-19 pandemic in a low to middle income country – A cross-sectional study

**DOI:** 10.1016/j.amsu.2021.102786

**Published:** 2021-09-04

**Authors:** Pirbhat Shams, Intisar Ahmed, Hunaina Shahab, Zehra Kadani, Aisal Khan, Marvi Shams, Yawer Saeed, Saira Bokhari, Aamir Hameed Khan

**Affiliations:** a Section of Cardiology, Department of Medicine, The Aga Khan University Hospital, Karachi, Pakistan; b Tabba Heart Institute, Karachi, Pakistan; c Department of Surgery, The Aga Khan University Hospital, Karachi, Pakistan

**Keywords:** Medical education, Virtual learning, e-learning, Cardiovascular medicine, Cardiology, Fellow-in-training, Post-graduate medical education, Academic, Simulators

## Abstract

**Background:**

COVID-19 pandemic has introduced us to a greater need of virtual learning platforms and has resulted in less clinical exposure for fellows-in-training. Virtual and simulator-based learning is not widely available in LMIC. It is imperative to analyze feedback of CV fellow-in-training regarding this mode of learning before large scale implementation.

**Methodology:**

This was an observational study conducted between July–August 2020. A multicentered survey was conducted. Survey questionnaire was disseminated to FIT (fellow-in-training) via Google Forms. The questionnaire contained a total of 24 questions about virtual and simulator-based learning during the pandemic.

**Results:**

A total of 68 FIT responded to the survey. The mean age was 29.9 years. There were 37% females and 63% males. Majority (75%) agreed that it was easier for them to reach for online sessions than physical sessions. 60% FIT were confident in asking questions or giving comments during the online sessions. 57.4% FIT felt it easier to go through cardiovascular imaging/illustrations via online platforms. 50% (34) were confident that if online sessions had to continue, they would have enough academic learning before they graduated from the program and 54.4% (37) wanted online sessions to continue even beyond the pandemic days. 37.5% (18 out of 48) agreed that the simulator-based teaching was helping them practice skills in times of less clinical exposure.

**Conclusion:**

COVID-19 pandemic has significantly impacted cardiovascular FIT learning curve because of less hands-on and lack of physical teaching sessions. LMIC have lack of robust e-learning platforms. Virtual learning is convenient for academic learning with growing acceptance amongst fellows. FIT from LMIC are less acquaint to simulator-based teaching and there is a need to invest in simulator-based cardiovascular teaching in LMIC.

## Highlights

• COVID-19 pandemic has affected cardiovascular FIT learning curve in terms of less clinical and procedure related exposure.
• Virtual education has started gaining acceptance in LMIC, but lack of wide availability is still the concern.

## 1 Introduction

COVID-19 pandemic has created huge training challenges in view of lack of in-person meetings and reduced bedside clinical teaching. This is especially true for cardiovascular Fellow-in-Training (FIT) [1]. Worldwide, learning mechanisms for health care workers (HCW) have been switched to online sessions in an effort to maximize social distancing and avoid exposures amongst HCW [2]. Virtual and simulator-based learning platforms are not widely available in Low to Middle Income Countries (LMIC) [3] and Asia harbors only 14% of institutes having access to e-learning medical education platforms [4]. It is prudent to analyze the feedback of FIT before implementing virtual cardiovascular teaching in a resource constraint setting. This pandemic has highlighted the importance of e-platforms which have emerged as the primary education tool in cardiovascular learning in Karachi which hosts three major institutes for cardiovascular training.

## 2 Methods

This was a multicentered survey conducted in Karachi. The study included three centers of cardiovascular training in the city. Google Form survey was circulated to the Program Directors (PD) and FIT via emails and/or WhatsApp. The survey included 24 questions regarding virtual and simulator-based learning experience during the pandemic. Participants included FIT from following sections: Adult cardiology, cardiothoracic surgery, and interventional cardiology.

Virtual learning methods included online Zoom/Teams sessions, video conference based interactive teaching (including CV imaging and electrocardiograms), online educational Quiz, journal clubs and grand rounds. Feedback was evaluated based on individuals’ responses to Google Form. The period of virtual learning for all responders was 3 months (April–June 2020). Responders were evaluated based on their response to the survey. The percentage of responders saying YES was calculated.

Ethical approval was obtained from the ethical review committee of the hospital. The work has been reported in line with the STROCSS criteria [5].This study has been registered with clinicaltrial.gov (UIN number: NCT04984564).

## 3 Results

A total of 68 FIT responded to the survey. The mean age was 29.9 years. There were 37% females and 63% males. Adult cardiology constituted 88.2% (60) of the responders, the remainder being cardio-thoracic surgery (8.8%) and interventional cardiology (2.9%). 94% (64) were concerned that COVID-19 pandemic was affecting their training due to less clinical and procedure related exposure. 85% (58) experienced increase in their stress level, 64.7% (44) felt that the pandemic was affecting their mental health and 77.9% (53) were afraid to contract COVID-19 while on work. 75% (51) agreed that it was easier for them to reach for online sessions than physical sessions. 60.3% (41) felt that they were not afraid of being judged for their questions and comments during the online sessions and 57.4% (39) thought that cardiovascular imaging/illustration teaching (including electrocardiograms and echocardiograms) had become easier due to the online platforms. 50% (34) were confident that if online sessions had to continue, they would have enough academic learning before they graduated from the program and 54.4% (37) wanted online sessions to continue even beyond the pandemic days. 53.7% (36) believed that Facebook based cardiovascular forums had increased their access to and reading time of cardiovascular literature. However, the same poll for Twitter could attract 16.2% (11) FIT. 76.5% (52) said that they missed the impact of physical sessions on their professional grooming. 37.5% (18 out of 48) agreed that the simulator-based teaching was helping them practice skills in times of less clinical exposure as this pandemic and 22.9% (11 out of 48) were neutral about it. However, 33.3% (16 out of 48) FIT responded that they had never experienced simulator-based teaching (Table 1.

**Table 1:** Cardiovascular fellow-in-training feedback on virtual learning experience during the pandemic.

Question	Percentage (%) of responders saying YES	Number of responders saying YES
COVID-19 pandemic is affecting my training with regard to less clinical and procedure related exposure	94	64
I feel increased stress during the pandemic	85	58
The pandemic is affecting my mental health	65	44
I am afraid of contracting the virus while on work	78	53
Online education attendance is easier for me	75	51
I am not judged for my questions during online sessions	60	41
I am confident I will graduate academically sound	50	34
Virtual learning should continue even after pandemic ends	54	37
Simulator-based teaching is useful for my training during the pandemic	38	18 out of 48
I do not have access to simulator-based training	33	16 out of 48
Facebook cardiovascular forums are useful for my clinical knowledge	54	36
Twitter cardiovascular forums (including Twitter Journal clubs) are useful for my clinical knowledge	16	11
COVID-19 pandemic has motivated me for more research	32	22
I miss physical academic meetings because they helped me in grooming professionally and in developing professional/personal relationships.	77	52
Virtual grand-rounds are as beneficial as in-person grand-rounds	28	19
If online education continues, I am confident I will have enough learning on graduation	50	34
Journal clubs make more sense now than ever before	38	26
ECG and other CV imaging learning has become easier	57	39
My concentration span is better in online sessions	39	27

## 4 Discussion

COVID-19 pandemic has introduced us to a greater need for virtual learning [6]. Worldwide, with lingering effects of pandemic, there was a transition to virtual learning platforms including schools, colleges, and universities. This included medical undergraduate and postgraduate training programs [7].

Academic hours are essential part of cardiovascular fellow-in-training (FIT) learning. These include core lectures, journal clubs, morbidity and mortality meetings and other scholarly activities. Transition to online cardiovascular learning has affected institutes and fellows worldwide [1]. The Accredited Council for Graduate Medical Education has recognized that reduction in hands-on experience during the pandemic will affect training quality of cardiology FIT and their future independent practices. Various institutes have recognized jeopardized cardiovascular training programs owing to the impact on clinical education and personal life [8]. Unfortunately, the transition to online system was not well-prepared for in third world countries. Trainees in LMIC face lack of technology and are being trained in techno-unfriendly educational systems. Through this study we looked at feedback of cardiovascular FIT about virtual learning experiences during the pandemic. We intended to know if or not the virtual system is applicable in days after the pandemic.

Our study revealed that majority of FIT experienced stress during the pandemic. Online learning platforms increased their convenience in reaching for online sessions and reduced their fear in asking questions during the sessions. However, only 50% were confident about the quality of academic learning using exclusive online academic system. One-third of our responders experienced reduced concentration span using e-platform and an equal third experienced better span. Rajab et al. looked at the challenges of online medical education system in Saudi Arab during COVID-19 pandemic. Of 208 responders, only 12% preferred exclusive online system, 25.5% wished for face-to-face instructions whereas 62.5% wanted combination of online and face-to-face learning system. The most encountered problems were communication (59%) followed by difficulty in student assessment (57.5%) and usage of technology tools (56.5%). Of note, 48% of responders felt increased stress or anxiety. 76% of responders showed willingness to integrate online method to current educational systems [9]. Moreover, a review of ten papers on online medical education from pre-COVID era tells us that the absence of institutional infrastructures and strategies, inadequate technical skills and time management were the main problems encountered in online medical education [10].

Our study showed that more than 90% of FIT were concerned that the pandemic was affecting their cardiovascular training. This is consistent with a survey-based study on interventional cardiology fellow training in New York metropolitan area during COVID-19 era which showed that reduction in cardiac catheterization lab (CCL) procedural volumes had impacted fellows’ training and their confidence [11]. The Society for Cardiovascular Angiography and Intervention surveyed interventional cardiology (IC) fellows and concluded that 49% of fellows believed that the pandemic was affecting their procedural skills and 65% experienced increased work stress. On the other hand, 97% of IC program directors responded positively with regard to end of training competency of their IC fellows [12]. A nation-wide survey of 997 cardiology FIT from the United States reported transition to virtual conferences (88%) and online education content (78%). 73% of this cohort showed interest in virtual lessons, tutorials, and simulators but 48% of the responders believed that cancellation of physical cardiovascular meetings and conferences had affected their advanced fellowship applications. This study also highlighted concerns of FIT regarding future job prospects keeping in view the economic impact of the pandemic [2]. The global concern about CV training quality is alarming and might bear repercussions over future individual practices of current FITs and their confidence.

Our study showed that only one third of fellows agreed to simulator-based learning being helpful. One-third of fellows did not have access to simulators and another one third were unsure about the benefits of this mode of learning. Majority of the responders felt Facebook forums as a good tool for CV learning which is not surprising keeping in view the growing usage of social platforms at international cardiovascular congresses for dissemination of information, for example, live tweets on late breaking trials [13]. Facebook is the most used free social platform for educational purposes in Pakistan and there is lack of widely available free educational platforms in the region and hence Facebook usage was inquired about in the survey.

It is important to differentiate between quality of pre-designed online courses and emergency remote training (ERT). ERT is a quick development of online education system in response to emergencies as this pandemic. In contrast to pre-designed online courses, the quality of ERT content can be questionable. It is a smart way to combat problems of medical education during the pandemic but not the ideal way given the unpreparedness. This is particularly true for LMIC. LMIC need to create robust institutional based systems and strategies for virtual education.

Despite the famed growth of virtual educational platforms, real hands-on learning is irreplaceable. The authors believe that amid this pandemic, a hybrid approach should be instituted for CV learning centers. This is particularly true for LMIC where there is lack of wide availability of digital gadgets, usually driven by economic constraints [14].

## 5 Limitations

This was a survey-based study. Individual responses were collected for a single point in time and did not reflect observation over short period of time, with lack of information about real-time trends. We were limited by the number of responders declining invitation to participate. The conclusion was applicable to a small number of responders and could not be generalized for FIT who did not respond to the survey.

## 6 Conclusions

COVID-19 pandemic has affected cardiovascular FIT learning curve in terms of less clinical and procedure related exposure. It also has significantly contributed to increase in stress level. LMIC have their own education and health challenges. If or not the colossal transition to online cardiovascular education system is sustainable or advisable, we needed to look at feedback of FIT from this part of the world. Considering our multi-centered study, we believe that virtual education has started gaining acceptance, but lack of wide availability and acceptance is still the concern. FITs are now comfortable and more interested to reach for virtual sessions. If or not, virtual cardiovascular learning is impacting the objective scores of performances, needs further research. Additionally, this brings us to the importance of establishing robust virtual and simulator-based learning platforms in LMIC for unfavorable days as these.

## Ethical approval

This was conducted as per ethical standards of the Aga Khan University Hospital Ethics Review Committee (ERC Number: 2020-5030-10817).

## Funding

No source of funding for this study.

## Consent

Informed consent was obtained at the start of questionnaire from all subjects. Once agreed to give consent, they were directed to main survey.

## Trial registry number

1. Name of the registry: clinicaltrial.gov.

2. Unique Identifying number or registration ID: UIN number: NCT04984564).

3. Hyperlink to your specific registration (must be publicly accessible and will be checked): https://clinicaltrials.gov/ct2/show/NCT04984564.

## Guarantor

Dr. Aamir Hameed Khan.

## Author contributions

Pirbhat Shams: Study conceptualization, design of the project, manuscript writing and revision.

Intisar Ahmed, Hunaina Shahab: Study conceptualization, design and literature review, draft revision.

Zehra Kadani, Aisal Khan and Marvi Shams: Data collection, analysis, literature review and manuscript writing.

Yawer Saeed, Aamir Hameed Khan and Saira Bukhari: Supervision from conceptualization, execution and to final manuscript editing.

## Provenance and peer review

Not commissioned, externally peer reviewed.

## Funding

No funding received for this research.

## Declaration of competing interest

None of the authors has any conflict of interest to declare.
